# MMP3 Is a Non-invasive Biomarker of Rejection in Skin-Bearing Vascularized Composite Allotransplantation: A Multicenter Validation Study

**DOI:** 10.3389/fimmu.2019.02771

**Published:** 2019-11-29

**Authors:** Branislav Kollar, Audrey Uffing, Thiago J. Borges, Andrey V. Shubin, Bruno T. Aoyama, Céline Dagot, Valentin Haug, Martin Kauke, Ali-Farid Safi, Simon G. Talbot, Emmanuel Morelon, Stéphanie Dakpe, Bohdan Pomahac, Leonardo V. Riella

**Affiliations:** ^1^Division of Plastic Surgery, Department of Surgery, Brigham and Women's Hospital and Harvard Medical School, Boston, MA, United States; ^2^Renal Division, Schuster Transplantation Research Center, Brigham and Women's Hospital and Harvard Medical School, Boston, MA, United States; ^3^Department of Molecular and Cellular Biology, Harvard University, Cambridge, MA, United States; ^4^Department of Transplantation, Nephrology and Clinical Immunology, Edouard Herriot Hospital, Hospices Civils de Lyon, Lyon, France; ^5^Department of Hand, Plastic and Reconstructive Surgery, Burn Trauma Center, BG Trauma Center Ludwigshafen, University of Heidelberg, Ludwigshafen, Germany; ^6^Department of Maxillo-Facial Surgery, Amiens University Hospital, Amiens, France

**Keywords:** acute rejection, biomarker, face transplantation, hand transplantation, vascularized composite allotransplantation

## Abstract

**Background:** There is unmet need for non-invasive immunomonitoring to improve diagnosis and treatment of acute rejection in vascularized composite allotransplantation (VCA). Circulating matrix metalloproteinase 3 (MMP3) was described as a candidate non-invasive biomarker to predict treatment response to acute rejection in clinical VCA. However, larger validation studies are yet to be reported to allow for more definitive conclusions.

**Methods:** We retrospectively measured MMP3 levels using ELISA in a total of 140 longitudinal serum samples from six internal and three external face transplant recipients, as well as three internal and seven external upper extremity transplant recipients. The control groups comprised serum samples from 36 kidney transplant recipients, 14 healthy controls, and 38 patients with autoimmune skin disease. A linear mixed model was used to study the effect of rejection state (pre-transplant, no-rejection, non-severe rejection (NSR), and severe rejection) on MMP3 levels.

**Results:** In VCA, MMP3 levels increased significantly (*p* < 0.001) between pre- and post-transplant no-rejection states. A further increase occurred during severe rejection (*p* < 0.001), while there was no difference in MMP3 levels between non-severe and no-rejection episodes. A threshold of 5-fold increase from pre-transplant levels could discriminate severe from NSR with 76% sensitivity and 81% specificity (AUC = 0.79, 95% CI = 0.65–0.92, *p* < 0.001). In kidney transplantation, the MMP3 levels were significantly (*p* < 0.001) elevated during antibody-mediated rejection but not during T-cell mediated rejection (TCMR) (*p* = 0.547). MMP3 levels in healthy controls and autoimmune skin disease patients were comparable with either pre-transplant or no-rejection/NSR episodes of VCA patients.

**Conclusion:** The results of this study suggest that serum MMP3 protein is a promising marker for stratifying patients according to severity of rejection, complementary to biopsy findings.

## Introduction

Vascularized composite allotransplantation (VCA) such as face and upper extremity (UE) transplantation can successfully restore form and function to patients with devastating injuries (1, 2). However, the transplantation of such complex tissues is associated with high rates of acute rejection (AR), reaching an incidence of more than 80% in the first postoperative year (3). Despite continuous progress in the VCA field, the diagnosis of AR is still limited to clinical presentation and skin biopsy assessment (4). Therefore, there is an unmet need for additional modalities to improve the diagnosis and treatment of AR, preferably in a non-invasive way.

The diagnosis of AR in VCA frequently poses challenges. In contrast to solid organ transplantation, the transplanted faces, and extremities can be exposed to external conditions such as variations in temperature, humidity, ultraviolet light, chemical/natural agents, minor injuries/traumas, and skin microbiota (5–7). These factors might amplify the adaptive immune response leading to rejection but also just mimic alloimmune injury through non-specific local inflammation. Indeed, many inflammatory conditions of the skin are histologically and clinically indistinguishable from AR (8).

The AR is traditionally diagnosed from skin biopsies according to Banff grading of skin-containing composite tissues (9). Interestingly, we observed in our center that around 80% of rejections which resolved with just topical therapy or adjustment of oral maintenance immunosuppression were scored by histology as grade 3, which is the same grade that usually leads to systemic administration of strong immunosuppression to treat rejection (10). Similar experience with treatment of AR was also reported in hand transplantation (11, 12). This lack of correlation of histopathological assessment with treatment response might be partially attributed to sampling bias or intra- and inter-observer variability of biopsy evaluation (13). Additionally, the clinical presentation of AR in face transplantation appears to evolve over time, leading to higher rate of subclinical rejection as well as subtler presentation of the traditional clinical signs of AR (e.g., erythema, edema, exanthema) at longer follow-up (14). Taken together, the decision to treat rejection is very complex and may significantly vary among different centers, in particular, if protocol biopsies are performed or not, which makes a standardized approach difficult. To provide the clinicians an additional modality in their decision making, we hypothesized that the severity of rejection could be measured through a non-invasive blood test as a surrogate marker of a more systemic immune system activation.

We recently reported circulating matrix metalloproteinase 3 (MMP3) as a promising non-invasive biomarker of severe rejection in a series of six face transplant recipients (10). However, more extensive validation studies have yet to be reported to allow for more definitive conclusions. Here, we provide longitudinal assessment of circulating MMP3 levels from 19 skin-bearing VCA recipients along with various control groups.

## Methods

### Study Design and Approval

In this retrospective multicenter cohort study, we measured serum levels of MMP3 from six Brigham and Women's Hospital (BWH) face transplant recipients, three BWH UE transplant recipients, three Lyon (France) face transplant, and seven Lyon (France) UE transplant recipients. All patients gave written informed consent to collect and process their blood samples as approved by the institutional review board (IRB) at BWH (Protocol #: 2010P000743) and at Amiens University Hospital, France (Protocol #: 2006-A00110-51). Acquisition of control serum samples from BWH kidney transplant recipients and healthy controls/autoimmune skin disease patients was approved under IRB protocol numbers 2017P000298 and 2018P001076, respectively.

### Serum Sample Collection

Blood samples from VCA patients were collected in parallel to the allograft skin biopsies at protocol visits as well as during suspected AR, as previously described (10). Serum samples from kidney transplant recipients were acquired through the BWH kidney transplant tissue repository. Collection of blood samples in the kidney transplantation group was always accompanied by a renal biopsy evaluated by Banff criteria (15). Blood samples from rejection episodes were taken before any treatment was initiated. Isolated serum was stored at −80°C until analysis with enzyme-linked immunosorbent assay (ELISA).

Serum samples from healthy controls (HC) and patients with autoimmune skin diseases were searched upon availability in the Partners Biobank to match BWH VCA patients in sex, age, and race. Partners Biobank is a large institutional repository of biological specimen from consented subjects which allows IRB approved investigators to search and select patient samples of interest for their research. Before requesting serum samples from Partners Biobank, the patients' phenotypes were verified through case-by-case chart review.

### Diagnosis of Rejection

Acute T-cell mediated rejection (TCMR) was diagnosed from 4-mm skin punch allograft biopsies in accordance to the Banff classification of skin-containing composite tissues with five grades from 0 to 4 (9). The biopsy grade was determined as consensus opinion of at least two experienced independent dermatopathologists. To diagnose antibody-mediated rejection (AMR), additional evidence of elevated levels of donor-specific antibodies (DSA) and accompanying clinical signs was necessary. An episode of rejection was defined as a biopsy showing Banff grade 2 or higher that required treatment. Since the authors do not treat Banff grade 1 rejection, biopsies with Banff grades 0 and 1 were deemed to be no-rejection (NR). Based on treatment response, the rejection episodes were retrospectively classified as non-severe and severe. Non-severe rejections (NSR) were managed by only adjustment of maintenance immunosuppression and/or topical therapy, while severe rejections (SR) required systemic administration of glucocorticoids and/or more potent drugs (e.g., thymoglobulin in case of steroid refractory rejection). More details about the treatment of AR can be found in previous publications (10, 16–20).

### MMP3 ELISA

Serum concentrations of MMP3 were measured by ELISA, using a commercially available kit (R&D Systems cat.# DMP300, Minneapolis, MN), according to the manufacturer's instructions, as previously described (10). The assay was reliable and reproducible, as evidenced by our intra- and inter-assay coefficient of variation of 2.3 ± 0.5% and 4.4 ± 2.5%, respectively.

### Statistical Analysis

We used a linear mixed model with random slope and intercept to study the effect of rejection state (Pre-TX, NR, NSR, and SR) on MMP3 levels in 140 longitudinal serum samples from 19 VCA patients. Rejection state was considered as fixed effect, individual patients as random effect. To meet normality assumptions, MMP3 levels were log-transformed and verified by visual inspection of Q-Q plots. Restricted maximum likelihood was used to estimate parameters. For every rejection state, estimated marginal means (EMM) of fitted models were calculated and the *p*-values from pairwise comparisons among the EMM were adjusted with Bonferroni correction. Independent control samples (kidney transplant recipients, HC, and autoimmune skin disease patients) were analyzed with parametric one-way ANOVA or non-parametric Kruskal-Wallis tests, as appropriate. Descriptive data of demographic and transplant characteristic between the VCA and kidney transplant cohorts were compared using the unpaired *t*-test, Chi-square test, or Fischer's exact test, as appropriate. All statistical tests were two-sided with a type 1 error rate of 0.05 to determine statistical significance. Statistical analysis was performed using IBM SPSS Statistics for Macintosh, Version 25.0 (IBM Corp, Armonk, NY). Continuous parametric variables are presented as mean and 95% confidence interval (CI) or standard deviation (SD). Continuous non-parametric variables are presented as median and interquartile range (IQR).

## Results

### VCA Patient Characteristics

Detailed VCA patient demographic information is presented in Table 1. The BWH cohort consisted of six face (patients 1–6) and three UE (patients 7–9) transplant recipients. The Lyon cohort included three face (patients 10–12) and seven UE (patients 13–19) transplant recipients. All patients received induction therapy with thymoglobulin and maintenance immunosuppression consisted typically of triple therapy with tacrolimus, mycophenolate, and prednisone. Steroids were completely weaned in patient 6, partially in patients 1–3 (reintroduction after fifth post-transplant year) and patient 7 received only seasonal (during winter) prednisone (7, 20). Due to a complicated rejection course, patient 5 received quadruple therapy with belatacept (21). In patient 11, a post-transplant lymphoproliferative disease five months after transplantation necessitated decreased maintenance immunosuppression (18). A total of 140 serum samples [including 24 previously published samples (10)] from 19 patients were analyzed for MMP3 levels: 16 pre-transplant samples, 78 no-rejection samples, 21 NSR samples, and 25 severe rejection samples. The pre-transplant serum was not available for patients 10, 11, and 17. Severe rejection occurred in all patients but 6, 7, 13, 16, and 19. NSR was encountered in all patients but 3, 8, and 10–12. The rejection episodes were predominantly cell-mediated, with exception of two late AMR episodes (patients 4 and 10) and two DSA positive rejections (patients 8 and 17). A detailed overview of all samples and respective MMP3 levels is presented in Supplementary Figure S1.

**Table 1:** Face and upper extremity transplant recipients' characteristics.

	**Patient 1**	**Patient 2**	**Patient 3**	**Patient 4**	**Patient 5**	**Patient 6**
Group	BWH face	BWH face	BWH face	BWH face	BWH face	BWH face
Date of transplant	05/2011	03/2011	04/2011	02/2013	03/2014	10/2014
Age at transplant (years)	D	A	A	C	B	B
Race	White	White	White	White	White	White
Mechanism of injury	Animal attack	Electrical burn	Electrical burn	Chemical burn	Ballistic trauma	Ballistic trauma
Extent of defect	Nose, cheek, eyelids, maxilla, lips	Forehead, nose, cheek, eyelids, lips	Forehead, nose, cheek, eyelids, lips	Forehead, nose, cheek, eyelids, lips	Nose, maxilla, mandible, lips	Nose, maxilla, mandible, lips
Allograft type	Full face	Full face	Full face	Full face	Partial face	Partial face
Induction agent	Thymoglobulin	Thymoglobulin	Thymoglobulin	Thymoglobulin	Thymoglobulin	Thymoglobulin
Maintenance immunosuppression	TAC/MMF/P	TAC/MMF/P	TAC/MMF/P	TAC/MMF/P	TAC/MMF/P/Bela	TAC/MMF
PRA (%)	0	68	0	97	22	32
DSA at transplant	Negative	Negative	Negative	Positive	Negative	Positive
HLA mismatch (A, B, DR)	5/6	4/6	4/6	5/6	5/6	5/6
CMV (Donor/Recipient)	Positive/Positive	Positive/Positive	Positive/Negative	Negative/Positive	Positive/Negative	Negative/Positive
EBV (Donor/Recipient)	Positive/Positive	Positive/Positive	Positive/Positive	Positive/Positive	Positive/Positive	Positive/Positive
Total ischemia time (hours)	2	4	2	3	3	1.5
	**Patient 7**	**Patient 8**	**Patient 9**	**Patient 10**	**Patient 11**	**Patient 12**
Group	BWH UE	BWH UE	BWH UE	Lyon face	Lyon face	Lyon face
Date of transplant	10/2011	10/2014	08/2016	11/2005	11/2009	06/2012
Age at transplant (years)	E	B	A	B	A	D
Race	White	White	White	White	White	White
Mechanism of injury	Septic shock	Septic shock	Ballistic trauma	Dog bite	Ballistic trauma	Vascular tumor
Extent of defect	Quadruple amputee	Quadruple amputee	Quadruple amputee	Nose, cheek, lips, chin	Lips, mandible	Lower eyelid, maxilla, tongue
Allograft type	Bilateral forearm	Bilateral upper extremity	Bilateral upper extremity	Partial face	Partial face	Partial face
Induction agent	Thymoglobulin	Thymoglobulin	Thymoglobulin	Thymoglobulin	Thymoglobulin	Thymoglobulin
Maintenance immunosuppression	TAC/MMF/P	TAC/MMF/P	TAC/MMF/P	Siro/MMF/P	TAC/MMF/P	TAC/MMF/P
PRA (%)	0	69	0	0	0	0
DSA at transplant	Negative	Positive	Negative	Negative	Negative	Negative
HLA mismatch (A, B, DR)	5/6	5/6	4/6	1/6	5/6	4/6
CMV (Donor/Recipient)	Negative/Negative	Negative/Negative	Negative/Negative	Negative/Positive	Negative/Negative	Negative/Negative
EBV (Donor/Recipient)	Positive/Positive	Positive/Positive	Positive/Positive	Positive/Positive	Positive/Negative	Positive/Positive
Total ischemia time (hours)	R4/L4	R4/L4	R4/L5	4	2	1.6
	**Patient 13**	**Patient 14**	**Patient 15**	**Patient 16**	**Patient 17**	**Patient 18**
Group	Lyon UE	Lyon UE	Lyon UE	Lyon UE	Lyon UE	Lyon UE
Date of transplant	01/2000	04/2003	02/2007	07/2008	07/2009	11/2012
Age at transplant (years)	B	A	A	A	A	B
Race	White	White	White	White	White	White
Mechanism of injury	Explosion	Crush	Electrocution	Burn	Explosion	Crush
Extent of defect	Bilateral upper amputee	Bilateral upper amputee	Bilateral upper amputee	Bilateral upper amputee	Bilateral upper amputee	Bilateral upper amputee
Allograft type	Bilateral upper extremity	Bilateral upper extremity	Bilateral upper extremity	Bilateral upper extremity	Bilateral upper extremity	Bilateral upper extremity
Induction agent	Thymoglobulin	Thymoglobulin	Thymoglobulin	Thymoglobulin	Thymoglobulin	Thymoglobulin
Maintenance immunosuppression	TAC/MMF/P	TAC/MMF/P	TAC/MMF/P	TAC/MMF/P	TAC/MMF/P	TAC/MMF/P/Eve
PRA (%)	0	0	0	0	0	0
DSA at transplant	Negative	Negative	Negative	Negative	Negative	Negative
HLA mismatch (A, B, DR)	5/6	4/6	4/6	3/6	5/6	6/6
CMV (Donor/Recipient)	Negative/Negative	Negative/Positive	Positive/Negative	Negative/Negative	Negative/Positive	Positive/Negative
EBV (Donor/Recipient)	Positive/Positive	Positive/Positive	Positive/Positive	Positive/Negative	Positive/Positive	Positive/Positive
Total ischemia time (hours)	R12.5/L13	R10/L10.5	R10/L10.5	R10/L8	R16/L20	R10/L10
	**Patient 19**					
Group	Lyon UE					
Date of transplant	11/2016					
Age at transplant (years)	D					
Race	White					
Mechanism of injury	Sepsis					
Extent of defect	Quadruple amputee					
Allograft type	Bilateral upper extremity					
Induction agent	Thymoglobulin					
Maintenance immunosuppression	TAC/MMF/P					
PRA (%)	0					
DSA at transplant	Negative					
HLA mismatch (A, B, DR)	6/6					
CMV (Donor/Recipient)	Negative/Negative					
EBV (Donor/Recipient)	Positive/Positive					
Total ischemia time (hours)	R5.5/L4.5					

*Age ranges (years): A = 20–30, B = 31–40, C = 41–50, D = 51–60, E = 61–70. Bela, belatacept; BWH, Brigham and Women's Hospital; CMV, cytomegalovirus; DSA, donor specific antibody; EBV; Epstein-Barr virus; Eve, everolimus; HLA, human leukocyte antigen; L, left extremity; MMF, mycophenolate; P, prednisone; PRA, panel reactive antibody; R, right extremity; Siro, sirolimus; TAC, tacrolimus; UE, upper extremity*.

### MMP3 Levels Increase After Transplantation and Peak During Severe Rejection

We used a linear mixed model to assess the effect of rejection state on MMP3 levels (Table 2). We found a significant increase (*p* < 0.001) of MMP3 levels from 6.97 ng/mL (95% CI: 4.71–10.33 ng/mL) to 24.1 ng/mL (95% CI: 17.34–33.42 ng/mL) between pre-transplant and post-transplant no-rejection state. An additional significant increase occurred during severe rejection (45.61 ng/mL, 95% CI: 31.56–65.92 ng/mL, *p* < 0.001), while there was no significant difference between the no-rejection and NSR (22.43 ng/mL, 95% CI: 15.32–32.59 ng/mL, *p* > 0.999) states. When looking at differences in the type of VCA, MMP3 dynamics were comparable between face and UE transplantation but statistical significance was not reached in the UE group alone (Table 2). Lastly, we did not find an association between the biopsy grades and MMP3 levels (Supplementary Figure S2).

**Table 2:** Effects of rejection state on MMP3 levels in linear mixed model.

**Cohort**	**Estimated marginal means**					**Pairwise comparisons**			
	**Rejection state**	**Mean (ng/mL)**	**df**	**95% CI lower bound (ng/mL)**	**95% CI upper bound (ng/mL)**	**Pre-TX vs. NR**	**NR vs. NSR**	**NR vs. SR**	**NSR vs. SR**
Face (*n* = 9, 75 serum samples)	Pre-TX	6.24	18.633	3.27	11.94	*p* < 0.001	*p* > 0.999	*p* < 0.001	*p* < 0.001
	NR	19.64	8.715	11.05	34.92				
	NSR	16.64	14.976	8.94	30.98				
	SR	41.12	11.133	22.7	74.31				
UE (*n* = 10, 65 serum samples)	Pre-TX	7.68	25.029	4.6	12.8	*p* < 0.001	*p* > 0.999	*p* = 0.253	*p* = 0.534
	NR	28.71	10.91	18.58	44.37				
	NSR	28.91	23.211	17.5	47.76				
	SR	45.82	31.587	26.43	79.62				
VCA (*n* = 19, 140 serum samples)	Pre-TX	6.97	45.306	4.71	10.33	*p* < 0.001	*p* > 0.999	*p* < 0.001	*p* < 0.001
	NR	24.1	20.353	17.34	33.42				
	NSR	22.34	38.863	15.32	32.59				
	SR	45.61	35.208	31.56	65.92				

*Linear mixed model analysis of 19 VCA patients (9 face transplants and 10 upper extremity transplants) was used to study the effect of rejection state on the MMP3 levels from 140 longitudinal serum samples. The p-values from pairwise comparisons among the estimated marginal means were adjusted with Bonferroni correction. MMP3 levels were backtransformed from logarithmic units to ng/mL. CI, confidence interval; df, degree of freedom; NSR, non-severe rejection; NR, no-rejection; Pre-TX, pre-transplant; SR, severe rejection; UE, upper extremity*.

### Severe Rejection Is Accompanied With at Least a 5-Fold Increase of MMP3 From Pre-transplant Levels

**Figure 1:**
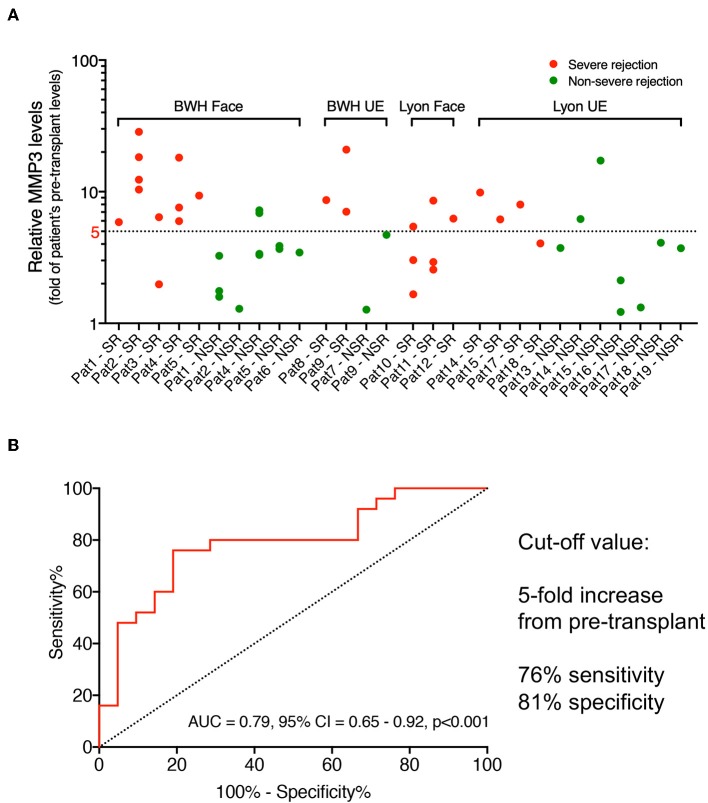
Cut-off values of MMP3 levels to predict treatment response. **(A)** For all VCA patients (x-axis), the fold-change from each patient's pre-transplant MMP3 levels (y-axis, logarithmic scale) was calculated for every severe (red dots) and non-severe (green dots) rejection episode. **(B)** Receiver operating characteristic (ROC) curve (AUC = 0.79, 95% CI: 0.65–0.92, *p* < 0.001) between severe (*n* = 25) and non-severe (*n* = 21) rejection episodes determined 5-fold change from pre-transplant levels as optimal cut-off value with 76% sensitivity and 81% specificity. AUC, area under the curve; BWH, Brigham and Women's Hospital; CI, confidence interval; UE, upper extremity; VCA, vascularized composite allotransplantation.

In order to predict the treatment response, we aimed to define a clinically relevant cut-off between the non-severe and severe rejection states. Considering the individual baseline differences of MMP3 levels between the patients, we calculated a fold-increase between the pre-transplant levels and rejection episodes for every patient (Figure 1A). In patients 10, 11, and 17 with no pre-transplant serum, we estimated the pre-transplant levels using the statistical information from linear mixed model analysis, which determined an average 3.46-fold increase of MMP3 levels between the pre-transplant and no-rejection states (EMM NR/EMM Pre-TX, Table 2). Accordingly, we divided the mean of all no-rejection samples from the patients 10, 11, and 17 by 3.46 to have a proxy of their individual pre-transplant levels. Finally, a receiver operating characteristic (ROC) curve analysis (AUC = 0.79, 95% CI: 0.65–0.92, *p* < 0.001) determined that at a cut-off value of 5-fold increase from pre-transplant levels, the separation between non-severe and severe rejection was achieved with 76% sensitivity and 81% specificity (Figure 1B).

### AMR in Kidney Transplantation Is Associated With Higher MMP3 Levels

**Figure 2:**
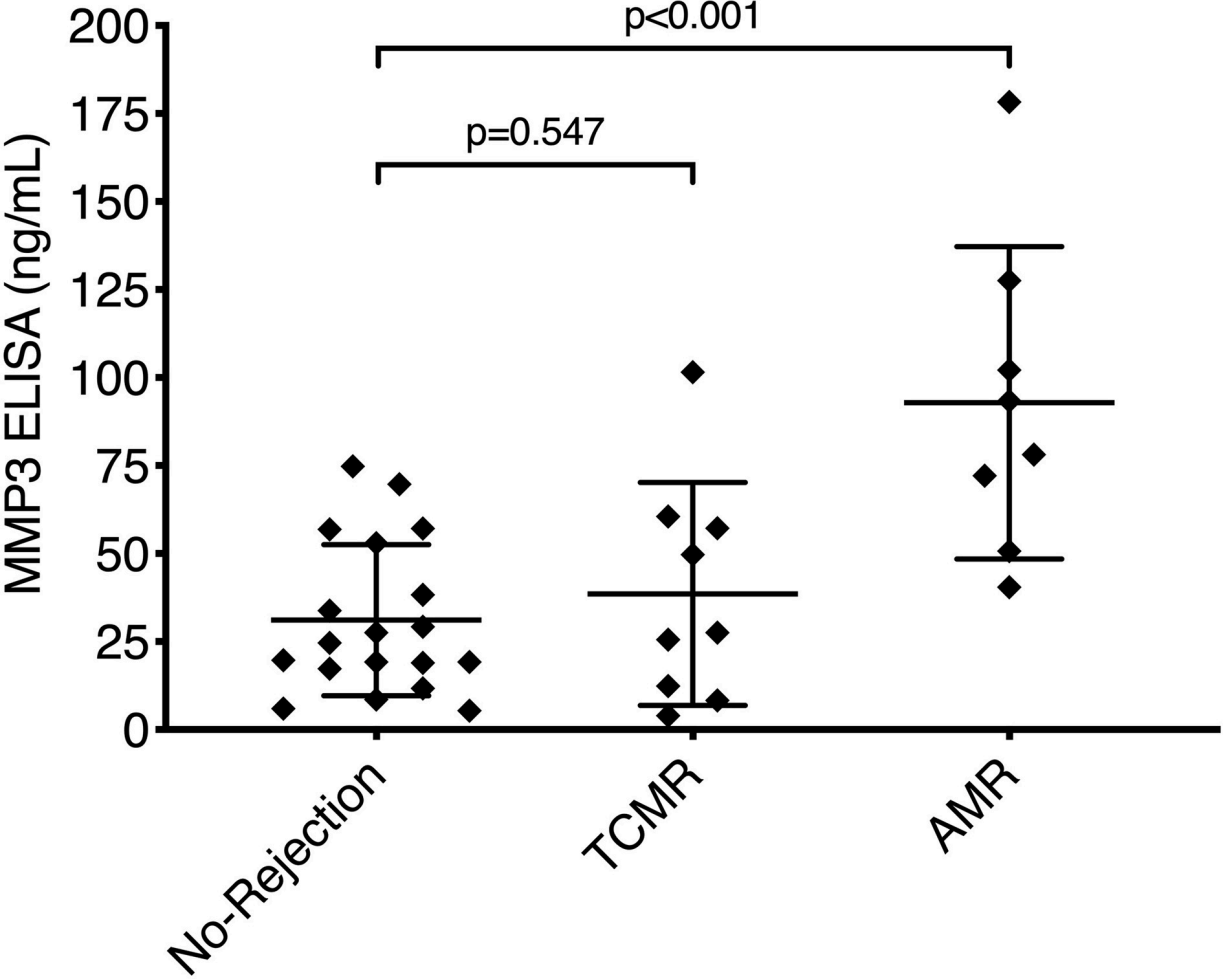
MMP3 levels in kidney transplantation. Serum levels of MMP3 from 36 kidney transplant recipients were measured during no-rejection (*n* = 19), TCMR (*n* = 9), and AMR (*n* = 8) episodes. There was a significant increase (*p* < 0.001) of MMP3 during AMR but not TCMR, as compared to no-rejection episodes. Data is presented as scatter dot plot showing every individual value. Mean and standard deviation are displayed as long and short horizontal lines, respectively. Statistical significance was evaluated with a parametric one-way ANOVA test followed by Holm-Sidak's *post-hoc* test. AMR, antibody-mediated rejection; TCMR, T-cell mediated rejection.

To evaluate MMP3 levels in a non-skin-bearing transplant, control serum samples from 36 available BWH kidney transplant patients were included: 19 represented no-rejection episodes, 9 represented TCMR episodes, and 8 represented AMR episodes. In the TCMR group, two, five, one, and one patients had biopsy grades of IA-IB, IB, IIA, and III, respectively. The kidney transplant groups' demographic and immunological characteristics are presented in Table 3. In the kidney transplant NR and TCMR groups, basiliximab was used as induction agent in 11% and 12% patients, respectively. All kidney transplant patients who had AMR had been induced with thymoglobulin (missing data for two patients). Importantly, there was not any statistically significant difference in neither induction nor maintenance immunosuppression treatment between kidney transplant (NR, TCMR, and AMR) and VCA cohorts (Table 3). The mean MMP3 levels did not significantly differ (*p* = 0.547) between patients with NR (31.1 ng/mL, SD: 21.24 ng/mL) and TCMR (38.53 ng/mL, SD: 31.66 ng/mL). However, there was a significant increase (*p* < 0.001) of MMP3 in patients with AMR (mean 92.84 ng/mL, SD: 44.35 ng/mL) compared to patients without rejection (Figure 2).

**Table 3:** Kidney transplant recipients' characteristics.

	**BWH kidney TX “NR” (*n* = 19)**	**BWH kidney TX “TCMR” (*n* = 9)**	**BWH kidney TX “AMR” (*n* = 8)**	**VCA (*n* = 19)**	***p*-value**
**Sex**					
Male	12 (63%)	5 (56%)	7 (88%)	14 (74%)	*0.73, #0.40, $0.63
Female	7 (37%)	4 (44%)	1 (12%)	5 (26%)	
**Age at TX (years, mean** **±** **SD)**	43.4 ± 12.4	52.1 ± 16.3	36.8 ± 14.2	36.9 ± 12.3	*0.11, #0.01, $0.98
**Race**					
White	14 (74%)	6 (67%)	6 (75%)	19 (100%)	*0.05, #0.03, $0.08
Black	5 (26%)	3 (33%)	2 (25%)	0 (0%)	
**Induction agent**					
ATG/Alemtuzumab	17 (89%)	7 (78%)	6 (75%)	19 (100%)	*0.49, #0.10, $0.99
Basiliximab	2 (11%)	2 (12%)	0 (0%)	0 (0%)	
Unknown	0 (0%)	0 (0%)	2 (25%)	0 (0%)	
**Maintenance immunosuppression**					
CNI	19 (100%)	8 (89%)	6 (75%)	18 (94%)	*0.99, #0.99, $0.20
MMF/MPA	19 (100%)	7 (78%)	6 (75%)	19 (100%)	*0.99, #0.10, $0.08
Azathioprine	0 (0%)	2 (22%)	1 (12%)	0 (0%)	*0.99, #0.10, $0.30
Sirolimus/Everolimus	0 (0%)	0 (0%)	2 (25%)	2 (11%)	*0.49, #0.99, $0.56
Prednisone	13 (68%)	8 (89%)	8 (100%)	18 (94%)	*0.09, #0.99, $0.99
Belatacept	0 (0%)	1 (11%)	0 (0%)	1 (6%)	*0.99, #0.99, $0.99
**PRA (%, mean** **±** **SD)**	20.3 ± 29.9	35.3 ± 36.4	34.6 ± 37.2	15.2 ± 29.8	*0.61, #0.13, $0.20
**DSA at TX**					
Positive	2 (10%)	2 (22%)	2 (25%)	3 (16%)	*0.99, #0.99, $0.27
Negative	16 (84%)	7 (78%)	3 (37.5%)	16 (84%)	
Unknown	1 (6%)	0 (0%)	3 (37.5%)	0 (0%)	
**HLA mismatch (A, B, DR)**					
<3/6	0 (0%)	1 (11%)	0 (0%)	1 (6%)	*0.99, #0.99, $0.99
≥3/6	14 (74%)	8 (89%)	6 (75%)	18 (94%)	
Unknown	5 (26%)	0 (0%)	2 (25%)	0 (0%)	
**CMV serostatus**					
D+/R+	8 (42%)	5 (56%)	3 (38%)	2 (11%)	*0.07, #0.06, $ <0.01
D+/R–	1 (6%)	2 (12%)	1 (12%)	4 (21%)	
D–/R+	4 (21%)	1 (11%)	0 (0%)	5 (26%)	
D–/R–	4 (21%)	1 (11%)	0 (0%)	8 (42%)	
Unknown	2 (10%)	0 (0%)	4 (50%)	0 (0%)	
**EBV serostatus**					
D+/R+	11 (57%)	7 (78%)	5 (62%)	17 (89%)	*0.07, #0.88, $0.37
D+/R–	1 (6%)	1 (11%)	0 (0%)	2 (11%)	
D–/R+	2 (10%)	0 (0%)	0 (0%)	0 (0%)	
D–/R–	1 (6%)	0 (0%)	0 (0%)	0 (0%)	
Unknown	4 (21%)	1 (11%)	3 (38%)	0 (0%)	
**Total ischemia time (minutes, mean** **±** **SD)**	676 ± 634	400 ± 466	1,191 ± 461	349 ± 273	*0.05, #0.71, $ <0.01
**Follow-up (months, mean** **±** **SD)**	16.8 ± 33	11.8 ± 10.4	37.8 ± 45.4	76.1 ± 45.2	*<0.01, # <0.01, $0.06

Data are presented as number (%) of patients, unless otherwise indicated. Variables were compared using the unpaired t-test, Chi-square test or Fischer's exact test, as appropriate. Significance values between groups: *VCA vs. NR, #VCA vs. TCMR, $VCA vs. AMR. AMR, antibody-mediated rejection; ATG, thymoglobulin; BWH, Brigham and Women's Hospital; CNI, calcineurin inhibitor; CMV, cytomegalovirus; D, donor; DSA, donor specific antibody; EBV; Epstein-Barr virus; HLA, human leukocyte antigen; MMF/MPA, mycophenolate mofetil/mycophenolic acid; NR, no-rejection; PRA, panel reactive antibody; R, recipient; SD, standard deviation; TCMR, T-cell mediated rejection; TX, transplantation; VCA, vascularized composite allotransplantation.

### Patients With Autoimmune Skin Disease Have Low MMP3 Levels

**Figure 3:**
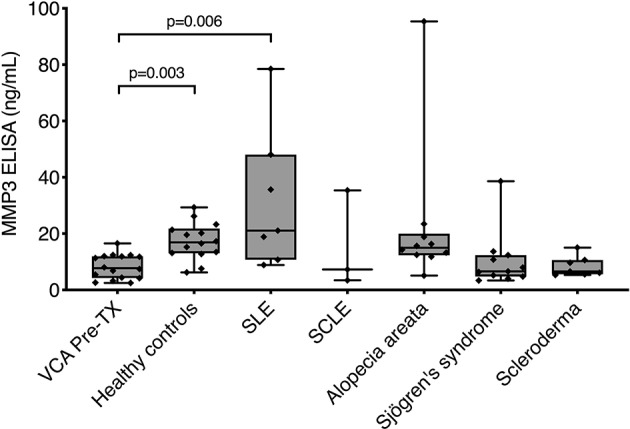
MMP3 levels in healthy controls and patients with autoimmune skin disease. Serum levels of MMP3 were measured in healthy controls (*n* = 14) as well as in patients with systemic lupus erythematosus (SLE, *n* = 7), subacute cutaneous lupus erythematosus (SCLE, *n* = 3), alopecia areata (*n* = 10), Sjögren's syndrome (*n* = 11), and limited cutaneous scleroderma (*n* = 7). When comparing to pre-transplant (Pre-TX) levels of VCA patients, there was a significant increase of MMP3 levels in healthy controls (*p* = 0.003) and SLE (*p* = 0.006) patients. There was no significant difference in MMP3 levels between the remaining groups. Data is presented as boxplots: in each boxplot, boxes delineate 1st (lower border) and 3rd (upper border) quartiles from the median (line within the box); whiskers represent minimum and maximum values. Individual patient values are presented as diamonds within the boxes. Statistical significance was evaluated with a non-parametric Kruskal-Wallis test followed by Dunn's *post-hoc* test.

To further study the specificity of increased circulating MMP3, we included control serum samples from 14 HC as well as 38 patients who experience non-alloimmune injury to the skin. The autoimmune skin disease group consisted of seven patients with systemic lupus erythematosus (SLE), three patients with subacute cutaneous lupus erythematosus, 10 patients with alopecia areata, 11 patients with Sjögren's syndrome and seven patients with limited cutaneous scleroderma. HC and patients with autoimmune skin disease were comparable in their demographic characteristics to the VCA cohort, although matching of the VCA cohort to patients with autoimmune skin disease was only partially possible due to the lower prevalence of these diseases in males (Tables 4, 5). Six out of seven patients in the SLE group received systemic immunosuppression, with prednisone and hydroxychloroquine being the most prevalent immunosuppressive drugs (57% of patients). Taking pre-transplant MMP3 levels of VCA patients as a reference (median 7.73 ng/mL, IQR: 4.25–11.95 ng/mL), we found higher levels of MMP3 in HC (median 16.9 ng/mL, IQR: 13.04–21.83 ng/mL, *p* = 0.003) and patients with SLE (median 21.05 ng/mL, IQR: 10.71–48.07 ng/mL, *p* = 0.006), but not in other patients with autoimmune skin disease (Figure 3). Nonetheless, MMP3 levels in HC and SLE patients did not reach severe rejection levels in VCA patients and were comparable to NR state of the VCA cohort (Table 2).

**Table 4:** Healthy controls' characteristics.

	**Healthy controls (*n* = 14)**	**VCA (*n* = 19)**
**Sex**		
Male	10 (71.4%)	14 (74%)
Female	4 (28.6%)	5 (26%)
**Age (years, mean** **±** **SD)**	38 ± 11.8	36.9 ± 12.3
**Race**		
White	14 (100%)	19 (100%)
Other	0 (0%)	0 (0%)

*Data are presented as number (%) of patients, unless otherwise indicated. BWH, Brigham and Women's Hospital; SD, standard deviation; VCA, vascularized composite allotransplantation*.

**Table 5:** Autoimmune skin disease patients' characteristics.

	**Alopecia areata (*n* = 10)**	**Sjogren's syndrome (*n* = 11)**	**Limited cutaneous scleroderma (*n* = 7)**	**Subacute cutaneous lupus erythematosus (*n* = 3)**	**Systemic lupus erythematosus (*n* = 7)**	**VCA (*n* = 19)**
**Sex**						
Male	7 (70%)	3 (27%)	1 (14%)	1 (33.3%)	1 (14%)	14 (74%)
Female	3 (30%)	8 (73%)	6 (86%)	2 (66.7%)	6 (86%)	5 (26%)
**Age (years, mean** **±** **SD)**	42.9 ± 12.7	49 ± 7.9	44 ± 9.88	44 ± 19	49.6 ± 9.8	36.9 ± 12.3
**Race**						
White	10 (100%)	11 (100%)	7 (100%)	3 (100%)	7 (100%)	19 (100%)
Other	0 (0%)	0 (0%)	0 (0%)	0 (0%)	0 (0%)	0 (0%)
**Systemic immunosuppression**						
CNI	0 (0%)	0 (0%)	0 (0%)	0 (0%)	0 (0%)	18 (94%)
MMF/MPA	0 (0%)	0 (0%)	3 (43%)	0 (0%)	1 (14%)	19 (100%)
Azathioprine	0 (0%)	0 (0%)	0 (0%)	0 (0%)	1 (14%)	0 (0%)
Sirolimus/Everolimus	0 (0%)	0 (0%)	0 (0%)	0 (0%)	0 (0%)	2 (11%)
Prednisone	0 (0%)	0 (0%)	0 (0%)	0 (0%)	4 (57%)	18 (94%)
Belatacept	0 (0%)	0 (0%)	0 (0%)	0 (0%)	0 (0%)	1 (6%)
Hydroxychloroquine	0 (0%)	2 (18%)	1 (14%)	1 (33.3%)	4 (57%)	0 (0%)

*Data are presented as number (%) of patients, unless otherwise indicated. BWH, Brigham and Women's Hospital; CNI, calcineurin inhibitor; MMF/MPA, mycophenolate mofetil/mycophenolic acid; SD, standard deviation; VCA, vascularized composite allotransplantation*.

## Discussion

Here, we demonstrate that by using a simple non-invasive blood test, the treatment of AR in VCA can be predicted with 76% sensitivity and 81% specificity. Circulating MMP3 is elevated during severe alloimmune injury in both face and UE transplantation as well as during AMR in kidney transplantation. On the other hand, MMP3 levels are lower during NSR and in patients with non-alloimmune injury to the skin. These findings show promise to advance biomarker-guided immunomonitoring in VCA.

The appropriate intensity of treatment response is crucial in VCA, since both conditions of over- and under-immunosuppression could have unwanted consequences in this non-life-saving transplant. However, the variety of differential diagnosis of rejection in VCA can make the appropriate diagnosis and treatment challenging. Numerous non-alloimmune conditions with high clinical and histological similarity with acute and chronic allograft injury were reported in the VCA literature. Cases of rosacea and erythema multiforme were shown in face (22) and UE transplantation (23), which eventually resolved with topical metronidazole and clobetasol treatment, respectively. In addition, clinico-pathologic findings resembling lupus-like lesions (interface dermal changes, hair follicle atrophy), scleroderma (skin fibrosis), and Sjögren's syndrome (lymphocytic sialadenitis) were demonstrated in various face transplant recipients (18, 24–26). To investigate whether MMP3 could distinguish these non-alloimmune skin injuries from clinically important rejection, we selected serum samples from patients with autoimmune skin diseases to serve as a control. We did not find an increase of MMP3 during the non-alloimmune skin conditions, with the exception of SLE. Increased levels of MMP3 in SLE have previously been described in patients with renal, joint and hematologic involvement (27). Since most SLE patients in our study received immunosuppressive therapy, suggesting a more severe disease phenotype, higher levels of MMP3 were to be expected. Overall, in contrast to a strong, systemic immune system activation during severe VCA rejection, it appears that NSR could be rather attributed to a more localized immune system response.

MMP3 is a proteolytic enzyme involved in normal and pathologic conditions such as tissue remodeling, cell signaling, or cancer (28). In transplantation, MMP3 has been linked to chronic transplant nephropathy (29) and bronchiolitis obliterans syndrome (30) after kidney and lung transplantation, respectively. Unfortunately, our study design does not allow us to imply whether MMP3 contributes to rejection in VCA or is just a consequence of tissue injury. Studies from our team showed that patients experiencing severe and repeated rejections have altered collagen expression in the dermis (18, 26). These findings suggest that MMP3 is involved in tissue repair, although mechanistic animal studies are needed to answer these open questions.

Although the results of this study are promising, there are couple of points that warrant more discussion. Notwithstanding that this is the most extensive non-invasive biomarker study in VCA to date, with nine face transplant patients representing 20% of the world-wide face transplant population (31), sample size remains modest and limits generalization of conclusions. Longitudinal data analysis in our study is particularly challenging since repeated measures were collected at irregular time-intervals, which precludes the use of traditional statistical tests. Linear mixed models present a solution since they can handle the above described issue and concomitantly adjust for within-subject correlation through random effects (32). To account for individual differences between patients, normalization to each patient's baseline (e.g., pre-transplant sample) was performed, rather than looking at absolute levels.

Furthermore, treatment of rejection in VCA is not as much standardized as in solid organ transplantation, mainly due to low case numbers and diverse immunosuppressive protocols between centers. Accordingly, in some VCA centers, grade 3 biopsies might be automatically treated with high dose systemic steroids, without a previous attempt of maintenance immunosuppression adjustment and/or topical therapy. In our center, we consider the clinical presentation in the assessment of the severity of rejection and in case a patient presents with mild clinical signs, less intense treatments are started first. Since superficial skin biopsy specimens are only a small and potentially biased representation of the whole allograft, additional diagnostic marker such as circulating MMP3 could contribute to a more objective and standardized diagnosis and treatment of rejection in VCA.

Overall, the results from this study indicate that serum MMP3 protein is a promising marker for stratifying patients according to severity of rejection, complementary to biopsy findings. Future steps include the design of a prospective randomized biomarker-guided trial, in which MMP3 levels may guide treatment decisions to optimize the clinical care of VCA recipients, reduce number of protocol biopsies and prevent complications related to over- or under-immunosuppression.

## Data Availability Statement

The datasets generated for this study are available on request to the corresponding author.

## Ethics Statement

The studies involving human participants were reviewed and approved by Partners Human Research Committee/IRB. The patients/participants provided their written informed consent to participate in this study.

## Author Contributions

BK and LR conceived the study and wrote the manuscript. BK, AU, and TB performed ELISA experiments. BK, AU, AS, BA, CD, VH, A-FS, MK, ST, EM, SD, BP, and LR participated in acquisition, analysis, and interpretation of data. BK and AU performed statistical analysis. All authors reviewed the manuscript critically for important intellectual content and gave final approval of the version to be submitted.

### Conflict of Interest

The authors declare that the research was conducted in the absence of any commercial or financial relationships that could be construed as a potential conflict of interest.

## Abbreviations

AMR: antibody-mediated rejection
AR: acute rejection
BWH: Brigham and Women's Hospital
ELISA: enzyme-linked immunosorbent assay
EMM: estimated marginal means
HC: healthy controls
NSR: non-severe rejection
NR: no-rejection
MMP3: matrix metalloproteinase 3
Pre-TX: pre-transplant
SR: severe rejection
TCMR: T-cell mediated rejection
UE: upper extremity
VCA: vascularized composite allotransplantation.
