# Supplementary material for: MMP3 Is a Non-invasive Biomarker of Rejection in Skin-Bearing Vascularized Composite Allotransplantation: A Multicenter Validation Study
Source: Front Immunol. 2019 Nov 29;10:2771. doi: 10.3389/fimmu.2019.02771 (PMC6897344; doi:10.3389/fimmu.2019.02771)
Supplement: Supplementary file 1 [file Data_Sheet_1.docx]

Supplementary Material

**Supplementary Figure S1.** Longitudinal individual circulating MMP3 levels of 19 VCA patients. The information on x-axis is given as time post transplantation, followed by rejection state and skin biopsy grade according to Banff classification. Severe and non-severe rejections are encircled in red and green, respectively. AMR, antibody-mediated rejection; G, Banff grade; M, postoperative month; NA, not available; NSR, non-severe rejection; NR, no-rejection; Pre-TX, pre-transplant; SR, severe rejection; W, postoperative week.

**Supplementary Figure S2.** Circulating MMP3 levels stratified according to Banff skin biopsy grades. MMP3 serum levels are presented as scatter dot plot in relation to the corresponding skin biopsy grades: Grade 0 (n=50), Grade 1 (n=25), Grade 2 (n=14) and Grade 3 (n=32). Linear mixed model analysis didn’t reveal any significant association of biopsy grades with circulating MMP3 levels. n.s., not significant.
